# Lutein‐20 Attenuates Proinflammatory Cytokines and Improves Antioxidant Enzyme Activity in an Animal Model of Alzheimer's Disease

**DOI:** 10.1002/alz70855_097666

**Published:** 2025-12-23

**Authors:** Adejoke Elizabeth Memudu, Peace Oluwabunmi Popoola, Amadi Ogonda Ihunwo, Neuroscience Research Group

**Affiliations:** ^1^ Edo State University Uzairue, Iyamho, Nigeria; ^2^ The Neuroscience Research Group of Anatomy Department Edo State University Uzairue, Edo State Nigeria, Iyamho‐Uzairue, Edo State Nigeria, Nigeria; ^3^ Edo State University Uzairue, Iyamho, Edo State, Nigeria; ^4^ School of Anatomical Sciences, Faculty of Health Sciences, University of the Witwatersrand, South Africa, Witwatersrand, Johannesburg, South Africa

## Abstract

**Background:**

Ethnopharmacology of plant based dietary compounds with antioxidant activity such as lutein‐20, needs to be evaluated in animal model of Alzheimer's disease (AD) to assess the antioxidant and inflammatory indexes., in addition to histological changes in the prefrontal cortex (PFC) and hippocampus (HP).

**Method:**

Twenty (20) adult male Wistar rats grouped into four (*n* = 5 viz; control, 20 mg/kg of Lutein‐20 treated group, AD model group exposed to 2 mg/kg of cadmium for 5 days, and the AD animal model group treated with 20 mg/kg of Lutein‐20 for 10 days. Serum was assayed for lipid peroxidation enzyme malondialdehyde (MDA), inflammatory marker total protein, pro‐inflammatory cytokines marker‐ tumour necrosis factor alpha (TNF alpha); antioxidant status of superoxidase dismutase (SOD), and Catalase (CAT) using spectrophotometric method. Statistical analysis was undertaken using one way ANOVA with significance set at *p* <0.05.

**Results:**

Our results demonstrated that cadmium significantly elevated markers of oxidative stress and pro‐inflammatory cytokines (MDA and TNF‐α), declined antioxidant enzyme activities (SOD, CAT, GSH), leading to neurodegeneration in the prefrontal cortex and hippocampus. However, administration of Lutein‐20 markedly reduced oxidative damage and suppressed neuroinflammatory responses through elevation of antioxidant enzyme activities resulting in a reduced disruption of the neuropil in the PFC and HP.

**Conclusion:**

Lutein‐20 attenuates pro‐inlnflammatory cytokines and improves antioxidant enzyme activity this underscores its neuroprotective potential as a therapeutic agent for neurodegeneration.

